# ISCHIOFEMORAL IMPINGEMENT: ASSESSMENT OF MRI FINDINGS AND THEIR RELIABILITY

**DOI:** 10.1590/1413-785220162406162686

**Published:** 2016

**Authors:** Ahmet Akça, Kadihan Yalçın Şafak, Ebru Dülger İliş, Zeki Taşdemir, Tamer Baysal

**Affiliations:** 1. Kartal Education and Research Hospital, Radiology Department, Istanbul, Turkey.; 2. Kartal Education and Research Hospital, Orthopedics and Traumatology Department, Istanbul, Turkey.

**Keywords:** Hip joint/pathology. Femur neck. Magnetic resonance ımaging/methods. Range of motion, articular. Reproducibility of results. Sensitivity and specificity

## Abstract

**Objective:**

To evaluate the Magnetic Resonance Imaging (MRI) findings and their validity in patients with ischiofemoral impingement syndrome (IFI) .

**Methods:**

We retrospectively analyzed 55 hips. MRI findings of 30 hips were consistent with IFI syndrome. Twenty five hips had no MRI findings consistent with IFI syndrome. We compared the ischiofemoral space (IFS), quadratus femoris space (QFS), ischial angle (IA) and femoral neck angle (FNA) between the age and gender matched groups. We also analyzed edema, fatty replacement and partial or total rupture of quadratus femoris muscle. Mann Whitney U test was used to compare the data.

**Results:**

We observed atrophy in eight, fatty replacement also in eight and edema in all of the quadratus femoris muscle. QFS (*p*<0.001) and IFS (*p*<0.001) were significantly lower in patients as compared to the control group. IA (*p*=0.012) and FNA (*p*=0.010) values were significantly higher in patients compared with the control group.

**Conclusion:**

MRI findings of IFI include narrowing of QFS and IFS and increase in IA and FNA. This condition should be kept in mind for patients with hip pain. Level of Evidence III, Retrospective Study.

## INTRODUCTION

Hip and groin pain is a common clinical condition and may affect patients of all ages. In an investigation by Christmas et al.^1^ 14.3% of adults aged 60 years and older reported significant hip pain on most days over the previous six weeks. Ischiofemoral impingement (IFI) syndrome can cause hip pain, which usually occurs in middle-aged to elderly women, being uncommon in males. However, IFI syndrome can affect both genders at all ages, ranging from 11 to 77 years. Bilateral hip involvement has been observed in 25-40% of patients.^2^ Despite the relevance of this clinical condition, IFI syndrome has been rarely described in the literature and remains a disputed subject.^3^ To our knowledge, IFI syndrome was first described in the literature by Johnson^4^ in three patients with hip pain after hip surgery. All three patients had hip pain and presented narrowing of the ischiofemoral space (IFS), which was relieved after lesser trochanter excision. More recently, Patti et al.^5^ described magnetic resonance imaging (MRI) findings of ischiofemoral narrowing and impingement in a patient with hip pain without a history of trauma or surgery, supporting IFI syndrome as a medical condition that clinicians, surgeons and radiologist should be aware of. Although the diagnosis of the IFI syndrome is complex, a number of smaller studies investigated the MRI findings of IFI syndrome in patients; most of these studies were case reports.^6^ ^-^ ^9^ However, if IFI syndrome is early diagnosed before any structural or pathological changes of the hip muscles and surrounding hip structures, it may be treated with nonsurgical methods.^9^ The aim of this study was to investigate the MRI findings and their reliability regarding IFI syndrome patients.

## MATERİALS AND METHODS

Institutional review board approval was obtained. Between June 2015 and February 2016 we included 20 consecutive patients (3 men, 17 women, age range 25-71, mean age 49.50±14.45 years old) with 30 hips (10 patients had bilateral signs and 10 patients had unilateral signs) showing MRI findings consistent with IFI syndrome and 17 patients (6 men, 11 women, age range 22-70 years, mean age 43.00±13.98 years) with 25 hips (eight patient had bilateral, nine patients had unilateral normal findings) with normal MRI findings within the IFS, QFS and quadratus muscle and no clinical symptoms compatible with IFI syndrome. Table 1 shows the patients' demographics. We retrospectively searched the patients who underwent hip MRI because of hip pain. The patients who had at least one of MRI findings of IFI syndrome, as previously described in the literature, (edema, fatty replacement, tear or muscle atrophy) were included in the study.^7^ Patients with osteoarthritis, history of trauma and surgery were excluded from the study. All patients underwent MRI using a 1.5 Tesla system (Philips Achieva 1.5 T system, Philips, Eindhoven, The Netherlands).The following parameters were applied: Axial T1-weighted (TR/TE 1180/11, matrix 320x126, slice thickness 7mm, FOV 444x233). Axial T2-weighted (TR/TE 3640/77, matrix 256x95, slice thickness 8.5mm, FOV 444x236. Coronal T1-weighted (TR/TE 524/22, matrix 320x185, slice thickness 6mm, FOV 413x500) and Coronal T2-weighted (TR/TE 3450/66, matrix 320x185, slice thickness 6mm, FOV 413x500).

**Table 1:** Patient Demographics.

Group	n	Gender (M/F)	Age (years old) Mean±St. Dev.
Patients	20	3/17	49.50±14.45
Controls	17	6/11	43.00±13.98

**Figure 1:**
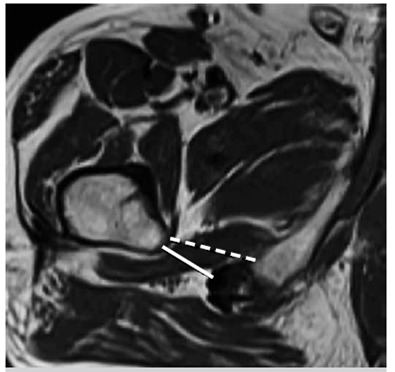
Measurements of quadratus femoris space (QFS) and ischiofemoral space (IFS). QFS is the smallest distance between hamstring origin and the lesser trochanter (solid line). IFS corresponds to the smallest distance between the lateral cortex of the ischial tuberosity and medial cortex of the lesser trochanter (dotted line).

**Figure 2:**
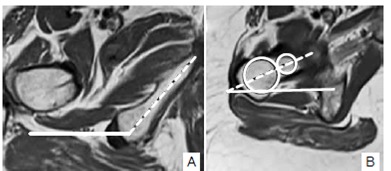
(A) Measurements of ischial angle (IA) and femoral neck angle (FNA). IA is the angle between the long axis of the ischiopubic ramus (dotted line) in reference to the horizontal plane (solid line); (B) Two circles were placed, one at the medial and one at the lateral aspect of the femoral neck, encompassing the entire space between the anterior and posterior femoral cortex, and a line passing through the midpoints of the circles was drawn. FNA is the angle between this line (dotted line) and the horizontal plane (solid line).

IFS distance was measured as the smallest distance between the lateral cortex of the ischial tuberosity and the medial cortex of the lesser trochanter. Quadratus femoris space(QFS) was measured as the smallest space for passage of the quadratus femoris muscle between the hamstring origin and the lesser trochanter, as previously described by Torriani et al.^6^ Figure 1 shows the QFS and IFS spaces and measurement. We also analyzed the QF muscle about the findings including edema, fatty replacement, tear and atrophy. We measured the ischial angle (IA) and femoral neck angle (FNA) as previously described by Bredella et al.^8^. Both angles were measured on axial images. The IA is the angle between the long axis of the ischiopubic ramus in reference to the horizontal plane (Figure 2a). FNA was measured at the level of the femoral neck that had no femoral head portion visible. At this level, two circles were placed, one at the medial and one at the lateral aspect of the femoral neck encompassing the entire space between the anterior and posterior femoral cortex, and a line passing through the midpoints of the circles was drawn. The FNA is the angle between this line and the horizontal plane (Figure 2b). 

Statistical analyses were performed using the SPSS software version 17. The variables were investigated using visual and analytic methods (Kolmogorov-Simirnov) to determine whether or not they are normally distributed. Since the measurements were not normally distributed; non parametric tests were conducted to compare these parameters. The Mann-Whitney U test was used to compare the QFS, IFS, IA and FNA between the patients and control group. A *p* value lower than 0.05 was considered a statistically significant result. The study was approved by the Ethics Committee, under number 89513307/1009/524. This is a retrospective study and the patients signed a Free and Informed Consent form for MRI tests.

## RESULTS

The mean QFS and IFS distance for patients were 7.05 ± 2.10 mm and 16.85±4.90 mm, respectively, while the mean QFS and IFS distance for controls were 16.32±3.64 mm and 26.98±7.90 mm, respectively. The mean IA and FNA values for patients were 131.57±5.04 mm and 27.94±8.86 mm respectively, while the mean IA and FNA values for controls were 127.26±3.57 mm and 22.18±10.01 mm, respectively. QFS (*p*<0.001) and IFS (*p*<0.001) distances were significantly lower in patients, as compared to the control group. IA (*p*=0.012) and FNA (*p*=0.010) values were significantly higher in patients, as compared to the control group. Table 2 shows the comparison and *p* values of the measurements.

**Table 2:** Measurements of QFS, IFS, IA and FNA.

Variables	Patients	Controls	p-value
QFS (mm)	7.05 ± 2.10	16.32 ± 3.64	p<0.001
IFS (mm)	16.85 ± 4.90	26.98 ± 7.90	p<0.001
IA (°)	131.57 ± 5.04	127.26 ± 3.57	p=0.012
FNA (°)	27.94 ± 8.86	22.18 ± 10.01	p=0.010

QFS: Quadratus femoris space. IFS: Ischiofemoral space. IA: Ischial angle. FNA: Femoral neck angle.

**Figure 3:**
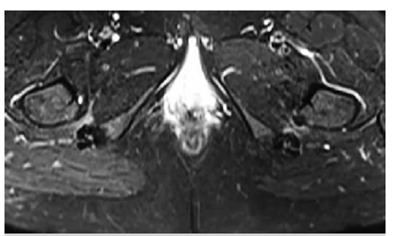
T2 weighted images showing the bilateral narrowing of quadratus femoris and ischiofemoral space, besides edema in the quadratus femoris muscle.

**Figure 4:**
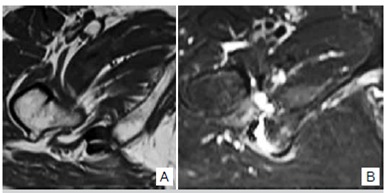
T1 weighted image (A) showing the atrophy and fatty replacement of QF muscle in a patient with IFI. (B) T2 weighted image of another patient with IFI showing rupture of QF muscle.

We observed atrophy in eight patients, fatty replacement also in eight patients and edema in all quadratus femoris muscle. One patient had rupture of quadratus femoris muscle. Figure 3 shows the NMR images of a patient with bilateral IFI. Figure 4a shows a patient with atrophy and fatty replacement of QF muscle, while Figure 4b shows a patient with rupture of QF muscle.

## DİSCUSSİON

IFI syndrome is a recently identified clinical condition characterized by hip pain, besides coexisting abnormalities of quadratus femoris muscle and narrowing of the space between the lesser trochanter of the femur and the ischial bone.^6^ The quadratus femoris muscle is a flat muscle originating from the lateral border of the ischial tuberosity and inserting into the quadrate tubercle and linea quadrata of the femur.^4^ This clinical condition may be associated with wasting or edema of the quadratus femoris muscle and may occur secondary to injury in other muscles controlling the movements of the hip, such as the hamstrings and gluteus medius.^10^ The underlying cause of IFI syndrome is uncertain. Acquired causes include intertrochanteric fractures, valgus producing intertrochanteric osteotomy and osteoarthritis, leading to superior and medial migration of the femur.^4^ Except acquired bony abnormalities, the cause of ischiofemoral narrowing can be congenital or positional.^11^ Patients with IFI syndrome complain of pain in the groin and/or buttock region.^5^ ^,^ ^12^ They may also describe hip pain that radiates to the knee.^12^ Often times, impingement symptoms can be reproduced upon adduction, extension and external rotation of the hip.^12^ However, Ali et al.^10^ reported that sometimes patients with IFI syndrome may be asymptomatic. It is especially important for radiologists to be aware of IFI syndrome, as it adds to the already long list of orthopaedic and non-orthopaedic conditions that may manifest itself as hip, groin and posterior thigh pain or asymptomatic radiologic findings.^13^ MRI determines inflammation and edema in the IFS and quadratus femoris, which is significantly different from an acute tear.^14^

Torriani et al.^6^ determined narrowing of the IFS and QFS in IFI syndrome patients with an abnormal quadratus femoris muscle. The mean IFS and QFS distances for control subjects in their investigation were 23±8 mm and 12±4 mm, respectively, while the mean IFS and QFS for IFI syndrome patients were 13±5 mm and 7±3 mm respectively. Khodair et al.^7^ found that the mean IFS and QFS distance for the healthy subjects were 20.7±1.4 mm and 15±0.9 mm, respectively, while the mean IFS and QFS distance for IFI syndrome patients were 15±1.8 mm and 8.8±1.3 mm, respectively. Bredella et al.^8^ found that the mean IFS and QFS distance for healthy subjects were 30.6±9.3 mm and 19.3±7.1 mm, respectively, while the mean IFS and QFS distances for IFI patients were 17.4±5.5 mm and 12.0±4.5 mm, respectively (*p*<0.0001). They determined that IA and FNA were greater in patients with IFI syndrome than control subjects, which may be predisposing factors for IFI syndrome. Patients with IFI syndrome had an increased IA, as compared to control subjects (130.6±0.9° *vs* 128.0±6.2°, *p*=0.004), which remained significant after normalizing for age and gender (*p*=0.01). Patients with IFI syndrome had an increased FNA as compared to control subjects (19.7±11.1° *vs* 15.5±12.1°, *p*=0.02), which remained significant after normalizing for age and gender (*p*=0.006). Singer et al.^15^ reviewed the imaging characteristics of IFI syndrome and performed a meta-analysis. They reported that cases of IFI patients had significantly smaller IFS (14.91±4.8mm *vs* 26.01±7.98mm, *p* < 0.0001) and QFS (9.57±3.7mm *vs* 15.97±6.07mm, *p* < 0.0001) as compared to controls. In our study, we found that QFS and IFS distances were significantly lower in IFI syndrome patients, as compared to the control group. QFS ranged from 3.09 to 9.8 mm, mean 7.05±2.10 mm and IFS ranged from 7.0 to 25.8 mm, mean 16.85±4.90mm. IA and FNA values were significantly higher in IFI syndrome patients, as compared to the control group. 

Khodair et al.^7^ found quadratus femoris muscle abnormalities in their study; eight (57.1%) of them showed diffuse muscle edema, three of them (21.4%) showed focal edema, two of them (14.3%) had partial tear, and one of them (7.2%) had diffuse muscle atrophy. Associated partial tear of hamstring tendon was found in one IFI syndrome patient (7.2%). No marrow edema or cystic changes were noted in the ischial tuberosity or lesser trochanter of any of IFI syndrome patients. Papavasiliou et al.^16^ reported a high incidence (62.5 %) of imaging findings of IFI in asymptomatic athletes. Özdemir et al.^17^ reported that bilateral IFS are asymmetrical in asymptomatic persons. They found ≥10 % of width difference between right and left IFS in approximately half of asymptomatic individuals. They reported that fatty infiltration and edema can be present in IFS in a small portion of the asymptomatic population, who also have narrower IFS than those without soft tissue MRI signal abnormalities. In our study, we determined atrophy in eight, fatty replacement also in eight and edema in all of the quadratus femoris muscles of IFI syndrome patients. We also identified the quadratus femoris muscle rupture in one IFI syndrome patient. 

## CONCLUSİON

We concluded that narrowing of QFS and IFS and increased IA and FNA in MRI scans are helpful in determining IFI syndrome. Our results were compatible with the literature. It is important for the radiologists to be aware of MRI findings of IFI syndrome, as it has been already added to the long list of orthopaedic and non-orthopaedic conditions that may manifest as hip, groin or posterior thigh pain. But it is also important to keep in mind that patients with MRI findings suggesting IFI syndrome may be asymptomatic. Although there are some studies evaluating the ischiofemoral impingement (IFI), MRI findings and their reliability remains unclear, because the same imaging findings have also been reported in asymptomatic populations. All patients included in this study complained of hip pain, who showed imaging findings consistent with IFI on MRI. The patients had no additional findings to explain hip pain. Thus, the diagnosis of IFI syndrome should be made on both clinical and radiological findings. We believe that larger scale MRI studies are needed to confirm the value of MRI findings in IFI syndrome.

## References

[1] Christmas C, Crespo CJ, Franckowiak SC, Bathon JM, Bartlett SJ, Andersen RE (2002). How common is hip pain among older adults? Results from the Third National Health and Nutrition Examination Survey. J Fam Pract.

[2] Tosun O, Algin O, Yalcin N, Cay N, Ocakoglu G, Karaoglanoglu M (2012). Ischiofemoral impingement: evaluation with new MRI parameters and assessment of their reliability. Skeletal Radiol.

[3] Kassarjian A (2008). Signal abnormalities in the quadratus femoris muscle: tear or impingement?. AJR Am J Roentgenol.

[4] Johnson KA (1977). Impingement of the lesser trochanter on the ischial ramus after total hip arthroplasty. Report of three cases. J Bone Joint Surg Am.

[5] Patti JW, Ouellette H, Bredella MA, Torriani M (2008). Impingement of lesser trochanter on ischium as a potential cause for hip pain. Skeletal Radiol.

[6] Torriani M, Souto SC, Thomas BJ, Ouellette H, Bredella MA (2009). Ischiofemoral impingement syndrome: an entity with hip pain and abnormalities of the quadratus femoris muscle. AJR Am J Roentgenol.

[7] Khodair SA, Ghieda UE, Elsayed AS (2014). Ischiofemoral impingement syndrome: Spectrum of MRI findings in comparison to normal subjects. Eg J Radiol Nuclear Med.

[8] Bredella MA, Azevedo DC, Oliveira AL, Simeone FJ, Chang CY, Stubbs AJ (2015). Pelvic morphology in ischiofemoral impingement. Skeletal Radiol.

[9] Lee S, Kim I, Lee SM, Lee J (2013). Ischiofemoral impingement syndrome. Ann Rehabil Med.

[10] Ali AM, Teh J, Whitwell D, Ostlere S (2013). Ischiofemoral impingement: a retrospective analysis of cases in a specialist orthopaedic centre over a four-year period. Hip Int.

[11] Ali AM, Whitwell D, Ostlere SJ (2011). Case report: imaging and surgical treatment of a snapping hip due to ischiofemoral impingement. Skeletal Radiol.

[12] Stafford GH, Villar RN (2011). Ischiofemoral impingement. J Bone Joint Surg Br.

[13] Shah A, Busconi B, DeLee JC, Drez D, Miller MD (2010). DeLee & Drez's orthopaedic sports medicine. Principles and practice.

[14] Tosun Ö, Çay N, Bozkurt M, Arslan H (2012). Ischiofemoral impingement in an 11-year-old girl. Diagn Interv Radiol.

[15] Singer AD, Subhawong TK, Jose J, Tresley J, Clifford PD (2015). Ischiofemoral impingement syndrome: a meta-analysis. Skeletal Radiol.

[16] Papavasiliou A, Siatras T, Bintoudi A, Milosis D, Lallas V, Sykaras E (2014). The gymnasts' hip and groin: a magnetic resonance imaging study in asymptomatic elite athletes. Skeletal Radiol.

[17] Maraş Özdemir Z, Aydıngöz Ü, Görmeli CA, Sağır Kahraman A (2015). Ischiofemoral Space on MRI in an Asymptomatic Population: Normative Width Measurements and Soft Tissue Signal Variations. Eur Radiol.

